# Fitness and Ecological Risk of Hybrid Progenies of Wild and Herbicide-Tolerant Soybeans With *EPSPS* Gene

**DOI:** 10.3389/fpls.2022.922215

**Published:** 2022-06-09

**Authors:** Laipan Liu, Li Zhang, Jianmei Fu, Wenjing Shen, Zhixiang Fang, Ying Dai, Ruizong Jia, Biao Liu, Jingang Liang

**Affiliations:** ^1^Key Laboratory on Biosafety of Nanjing Institute of Environmental Sciences, Ministry of Ecology and Environment, Nanjing, China; ^2^State Environmental Protection Scientific Observation and Research Station for Ecology and Environment of Wuyi Mountains, Nanjing Institute of Environmental Sciences, Ministry of Ecology and Environment, Nanjing, China; ^3^Hainan Key Laboratory for Biosafety Monitoring and Molecular Breeding in Off-Season Reproduction Regions, Sanya, China; ^4^Development Center of Science and Technology, Ministry of Agriculture and Rural Affairs, Beijing, China

**Keywords:** glyphosate-tolerant soybeans, gene flow, hybrid progeny, relative fitness, ecological risk

## Abstract

Exogenous genes of transgenic crops are usually transferred to their wild-type relatives through pollen-mediated gene flow, which may change the ecological fitness and ability to invade wild populations, resulting in the weeding of wild plants and other unpredictable environmental impacts. In this study, the F_1_ generation of herbicide-resistant soybeans and wild soybeans was obtained by artificial pollination, F_2_ generation seeds were obtained by self-crossing, and the fitness of the parents and their F_1_ and F_2_ generations were tested. The foreign protein *EPSPS* was expressed normally in the hybrid between transgenic and wild soybeans; however, the protein expression was significantly lower than that in transgenic soybeans. The fitness of the F_1_ hybrid between transgenic and wild soybeans was significantly lower than that of its parent. Compared with those of the wild soybeans, the F_2_ generation soybeans improved in some fitness indices, while the emergence rate, pollen germination rate, and number of full seeds per pod, pods per plant, and full seeds per plant did not significantly differ. The aboveground biomass and 100-seed weight of the F_2_ generation were higher than those of wild soybeans. Fitness among the F_2_-negative plants, homozygous, and heterozygous positive plants did not significantly vary. Improved fitness and presence of foreign genes in the F_2_ soybean were not significantly correlated. As the F_2_ generation of transgenic and wild soybeans had no fitness cost and the flowering stage were overlapped, the foreign gene might still spread in the wild soybean population.

## Introduction

The commercial application of genetically modified crops has resulted in significant economic gains (Pellegrino et al., 2018). At the same time, the biosafety of genetically modified crops has attracted widespread attention, especially the biological effect of the transfer of foreign genes from genetically modified crops to their wild relatives, which is a research hotspot, as well as the possible ecological risks through pollen-mediated gene flow (Snow et al., 2005; Lu and Yang, 2009; Ellstrand et al., 2013). The insertion of foreign genes gives transgenic crops the ability to withstand adversity; hence, when foreign genes are transferred to wild relatives, it is likely to change the ecological fitness and invasive ability of wild populations, resulting in the weeding of wild plants or other unpredictable environmental impacts (Snow et al., 1999; Lu, 2013; Lu et al., 2016). *EPSPS-*transgenic rape (*Brassica napus* L.) emerged through hybridization between rape and wild turnip (*Brassica rapa* L.) in Argentina. Gene flow occurs, and because of the strong selective usage of glyphosate herbicides in farmland weeding, the existence and expansion of this glyphosate-resistant turnip in farmlands have become increasingly intense in recent years (Pandolfo et al., 2018). Soybeans are not only one of the most important food crops, but also one of the earliest and most widely used genetically modified crops (ISAAA, 2019). China is the main distributor of wild soybeans worldwide. The foreign genes in transgenic soybeans may drift into wild soybeans, remaining and spreading throughout the wild soybean natural population for a long time, thus posing an ecological risk to the biodiversity of wild soybeans (Snow et al., 1999; Song et al., 2004; Lu, 2015).

At present, research on gene flow frequency and expression of transgenes escaping to wild related species is relatively well established. However, there is a lack of research on the changes in fitness and long-term ecological effects of different transgenes (including insect-resistant and herbicide-resistant transgenes) on wild related species after escape. Fitness is reflected by a series of nutritional and reproductive growth indicators, which are key to the biosafety of transgenes (Warwick et al., 2009). When a wild population acquires a foreign gene with strong selection advantage and is exposed to related selection pressures (such as herbicide spraying, pest attack, or drought/salt stress), it is likely to show strong fitness and evolutionary potential under natural selection, which may lead to harmful environmental consequences, such as additional weeding or increased invasive ability (Burke and Rieseberg, 2003; Ellstrand, 2003; Lu and Snow, 2005; Ellstrand et al., 2013). Foreign genes with no significant effect on fitness are expected to persist in the population, while those that increase the cost of fitness are expected to be removed from the population unless there is persistent gene flow (Ellstrand, 2003). The *EPSPS* gene can endow plants with glyphosate tolerance. In the presence of glyphosate herbicides, the fitness of wild species expressing the *EPSPS* gene was significantly higher than that of the original wild populations; in the absence of glyphosate herbicides, the *EPSPS* gene had similar effects on wild soybeans (Guan et al., 2015; Kan et al., 2015), wild rice (Wang et al., 2014), and *Arabidopsis thaliana* (Beres et al., 2018).

China is a large consumer of soybeans and imports a large quantity of *EPSPS*-transgenic soybeans every year; scientific and technological circles are calling for independent research and industrial-scale development of genetically modified soybeans. However, the relevant national authorities need scientific and technical support in making decisions on whether to approve the industrialization of transgenic soybeans and in managing the resulting environmental risks (especially controlling the introduction of foreign genes into wild soybeans). To date, there have been four studies conducted on the fitness of hybrid progeny between *EPSPS-*transgenic and wild soybeans, of which one study showed that the fitness of F_1_ hybrids of them in different regions was lower than that of wild soybeans (Liu et al., 2021). The results of the remaining three showed that, compared to wild soybeans, the fitness of those expressing the *EPSPS* gene did not change significantly in the absence of glyphosate and was even significantly higher than that of wild soybeans in some fitness parameters, indicating that wild soybeans expressing the *EPSPS* gene may be more invasive than their wild soybean parents (Guan et al., 2015; Kan et al., 2015; Yook et al., 2021). Therefore, it is impossible to draw a consistent conclusion about the fitness of hybrid offspring of genetically modified and wild soybeans.

In this study, transgenic and wild soybeans collected in the field were used to establish a hybrid progeny system with a foreign gene background to simulate the natural state of soybeans after foreign gene transfer in the natural environment. By testing the fitness of the parents and their hybrid F_1_ and F_2_ generations, we predicted evolutionary potential of foreign genes after transferring to wild related species and the possible long-term ecological risks. Because of the strict regulations on genetically modified crops by the Chinese government, soybeans containing foreign genes cannot be planted in fields; therefore, the experiment was carried out in a greenhouse.

## Materials and Methods

### Materials

In this study, genetically modified soybeans, GTS40-3-2 obtained from the fence and railway track of the processing plant of Hebei Sanhe Huifu Grain and Oil Food Production Co., Ltd. during the field investigation of genetically modified soybean in 2005 were crossed with the Jiangsu local soybean variety, Su Dou 12, and the resulting plants were screened four times to obtain homozygous transgenic soybean with glyphosate resistance. Wild soybean seeds were collected from Zhengzhou City, Henan Province, China, in 2015. *EPSPS-*transgenic soybeans and wild soybeans were planted in greenhouses in 2017. F_1_ seeds were obtained by artificial pollination with *EPSPS*-transgenic soybeans as the male parent and wild soybeans as the female parent, and F_1_ generation soybeans were planted in a greenhouse in 2018, with F_2_ seeds obtained by self-crossing.

### Location

The experiment was carried out under isolated conditions in the greenhouse of the Nanjing Institute of Environmental Science at the Ministry of Ecological Environment, and all the experimental materials were planted in the same soil and managed equally. After natural air-drying, grinding, and sieving, the soil samples were sent to the Nanjing Institute of Soil Research, Chinese Academy of Sciences for the determination of physical and chemical properties. The details of the soil in 2018 and 2019 are shown in Table 1.

**Table 1:** Physicochemical property of soils used for the tests in 2018 and 2019.

Year	Organic matter (g/kg)	Total nitrogen (g/kg)	Total phosphorus (g/kg)	Total potassium (g/kg)	Available phosphorus (mg/kg)	
2018	39.3	2.6	1.8	19.2	46.8	165.5
2019	42.1	2.7	1.9	21.1	47.9	172.7

### Method

In 2017, 15 *EPSPS*-transgenic soybean seeds were sown on May 10, 20, and 30, and 15 wild soybean seeds were sown on May 20, with one seed per pot. At the flowering stage (July 15–25, 2017), *EPSPS-t*ransgenic soybean pollen was collected for artificial pollination of the wild soybean; a total of approximately 100 flowers were pollinated successfully, and 136 full F_1_ seeds were obtained after the wild soybean plants matured.

On May 26, 2018, 120 F_1_ seeds, 60 EPSPS transgenic soybean seeds, and 60 wild soybean seeds obtained in 2017 were randomly selected. Each material was randomly divided into three groups and planted under the same soil and management conditions. Thirty soybean seedlings growing normally were randomly selected from each experimental set. The number of wild soybeans was replanted to 30 plants.

On May 26, 2019, F_2_ seeds obtained in 2018 (150 pieces of cut episperm seeds were divided into three groups on average, and 150 pieces of uncut episperm were divided into three groups on average), *EPSPS*-transgenic soybean seeds (60 pieces were divided into three groups on average), and wild soybean seeds (60 pieces of skin were cut and divided into three groups on average, and 60 pieces of uncut skin were divided into three groups on average) were planted under the same soil and management conditions, and the germination rate was recorded and counted.

### Determination of Biological Characteristics of Soybeans

#### Detection of Foreign Gene Genotypes in F_2_ Generation

Transformant-specific primers and probes were designed for real-time fluorescence quantitative PCR and digital PCR according to the transgenic transformant-specific sequence obtained by analysis. The specific sequences are listed in Table 2.

**Table 2:** Sequence of primers and probes used for copy number determination of genetically modified soybean.

Target sequence	Primer	Primer sequence (5′-3′)	Length
Lectin	Lectin-F	GCCCTCTACTCCACCCCCA	118 bp
	Lectin-R	GCCCATCTGCAAGCCTTTTT	
	Lectin-P	FAM AGCTTCGCCGCTTCCTTCAACTTCAC BHQ1	
GTS40-3-2	40-3-2-Q-1F	TTCATTCAAAATAAGATCATACATACAGGTT	84 bp
	40-3-2-Q-2R	GGCATTTGTAGGAGCCACCTT	
	40-3-2-Q-1P	FAM-CCTTTTCCATTTGGG-BHQ	

*GTS 40-3-2 transformant-specific sequence GenBank number: HW479040.*

Fresh leaves from 120 F_2_ soybean plants were randomly selected at the 3–4-leaf stage. Genomic DNA was extracted and purified using the DNeasy® Plant Mini Kit (QIAGEN, Germany) to determine the quality and concentration of DNA (NanoDrop 1,000).

##### TaqMan Real-Time PCR

The TaqMan real-time PCR was carried out on an ABI 7900HT thermocycler (Applied Biosystems, United States) with a reaction volume of 25 μl: 12.5 μl HR qPCR Master Mix (Huirui biotechnology, China), 0.4 μl F/R primer (10 μm/l), 0.2 μl #149 probe (10 μm/l), or TaqMan probe, 5 μl genomic DNA, and double distilled water to add to 25 μl. The PCR cycling program is: an initial denaturation at 95°C for 10 min, followed by 45 cycles of 95°C for 15 s and 60°C for 1 min. The fluorescence signal was monitored at the annealing step of each cycle. The real-time PCR data were collected and analyzed using SDS 2.4 software (Applied Biosystems, United States). Each reaction was repeated three times, and each time with three parallel reactions.

Using the difference in the standard Ct value from quantitative PCR amplification of lectin and the value for genetically modified transformants, we can determine whether the sample contains genetically modified transgenic components. This judgment is based on the following:

Positive results: exogenous Ct value <35; Ct value of lectin gene <35.

Negative results: no exogenous amplification, no Ct value, Ct value of lectin gene <35.

Reproducibility test for suspicious samples: For samples with exogenous Ct value >35 and Ct value <35, the DNA was re-purified and confirmed by amplification.

##### ddPCR

ddPCR reactions were carried out on a QX200 ddPCR system (Bio-Rad, Pleasanton, CA) in a reaction volume of 20 μl. Each ddPCR was composed of 10 μl 2 × ddPCR Supermix for probes (Bio-Rad, Pleasanton, CA), 0.4 μl forward and reverse primers, 0.2 μl TaqMan probe, 1 μl genomic DNA, and double distilled water to add to 20 μl. Droplets were generated in the emulsification device of the QX200 droplet generator (Bio-Rad, Pleasanton, CA). Water-in-oil emulsions were then transferred to a 96-well plate and heat-sealed with a pierceable foil seal. The 96-well plate was placed onto a T100 thermal cycler (Bio-Rad, Pleasanton, CA), and PCR amplifications were performed using the following program: an initial denaturation of 10 min at 95°C, 40 cycles of 15 s at 95°C and 1 min at 57.7°C, and a final extension of 10 min at 98°C. After PCR amplification, the 96-well PCR plate was transferred onto the QX2000 Droplet Reader to read the fluorescence signal and automatically analyzes the number of positive and negative droplets using the QuantaSoft software. By setting a fluorescence amplitude threshold, the positive droplets with amplified target DNAs were distinguished from the negative droplets with below-threshold fluorescence signals. For the data to be eligible for further analysis, the number of droplets detected by the QuantaSoft software has to be more than 8,000 per 20 μl. Each reaction was repeated three times, and each time with three parallel reactions. The theoretical value of the copy number ratio of homozygotes was 1 and that of heterozygotes was 0.5.

#### Detection of *EPSPS* Protein Content

All surviving soybean leaf samples were collected at the 3–4-leaf stage (uncertain date), and one complete leaf was collected from the top part of each plant. Immediately after the leaves were isolated, they were placed in a sealed centrifuge tube and stored in a liquid nitrogen tank. After being sent to the laboratory, the samples were stored in a refrigerator at −70°C.

Twenty transgenic soybeans and hybrid F_1_ were collected, and 20 phenotypes of hybrid F_2_ were selected and determined using the EnviroLogix (United States) *EPSPS* enzyme-linked immunosorbent assay kit (ENVIROLOGIX, QualiPlate Kit for CP4 *EPSPS*). The detection process was carried out according to the manufacturer’s instructions. The absorbance of the samples was determined using an enzyme-labeling instrument (TECAN Infinite M2000, Switzerland). The content of *EPSPS* protein in the sample was calculated according to the absorbance of the sample and the standard curve; then, the content of *EPSPS* protein in the leaves was calculated (μg/g).

### Determination of Growth and Development Indicators

#### Emergence Rate

The emergence rate of soybean for each material was calculated after soybean emergence, by calculating the seedling emergence and sowing number.

#### Growth Period

After the soybean materials were planted, the growth was observed every day, the date of the start of each growth period was recorded, and the duration of each growth period was calculated. The statistical growth period included germination, seedling, branching, budding, early flowering, full flowering, podding, and mature stages.

#### Aboveground Biomass

After the soybeans were fully matured, 10 soybean materials were randomly selected to measure the aboveground biomass. The soybean plants were cut off from close to the surface, allowed to dry naturally to a constant weight, and weighed (PB602-N, Mettler Toledo).

### Determination of Reproductive Index

#### Pollen Germination Rate

In the R2 stage (full flowering stage), the flower buds were selected from the normally developed plants from 9:00 to 10:00 in the morning, and the pollen color was recorded. The buds were removed with tweezers and placed in a clean centrifuge tube. After sending them to the laboratory, they were immediately placed in the culture medium, and after 2 h, pollen germination was observed using a Leica DM750 microscope (Nikon Corporation, Japan). Germination occurs when the length of the pollen tube exceeds the diameter of the pollen grain. The constitution of the culture medium was as follows: sucrose concentration, 19.2%; Gibberellin A3 concentration, 68.9 mg/l; initial pH, 6.47; boric acid concentration, 0.015%; calcium chloride concentration, 0.05%; and PEG-4000, 7.5%.

Four flowers were selected from each experimental set, three visual fields were randomly selected from each experimental treatment, and no less than 50 pollen grains were counted in each visual field. Using the number of germinated and non-germinated pollen grains, the germination rate was calculated as follows: germination rate = quantity of germinated pollen/total quantity of pollen × 100%.

#### Soybean Seed Index

After the soybean was fully matured, the number of pods per plant, full seeds per pod, full seeds per plant, and the weight of 100 seeds per plant were tallied (100 seeds per plant were randomly selected and 10 seeds per plant were tested).

### Data Analysis

The amount of *EPSPS* protein was expressed as the amount per gram of leaf (μg). LSD-T test (least significant difference, LSD) and Tukey multiple comparison methods were used to compare the expression levels of *EPSPS* protein in transgenic soybean, hybrid F_1_ generation, and F_2_ generation homozygous and heterozygous positive plants as well as to compare the fitness traits of transgenic, wild, F_1_, and F_2_ soybeans. The relative fitness was calculated by dividing the emergence rate of soybean of each set in the same experimental year by the emergence rate of wild soybean in the same year, and the calculation methods for aboveground biomass, pollen germination rate, number of full grains per plant, and 100-grain weight were the same. The relative fitness of each index was added to the mean value to calculate the comprehensive fitness index.

LSD analysis was used to analyze the correlation between the fitness index, hybridization, and protein expression. The specific calculation methods are as follows: For hybridization—the three phenotypes of hybrid F_2_ generation were classified into one group and wild soybean plants were classified as one group, and if the difference was significant, the variation of fitness was significantly related to hybridization; if it was not significant, the variation of fitness was unrelated. For protein expression, F_2_ negatives were classified into one group, and F_2_-positive homozygotes and heterozygotes, into another group; if the difference was significant, the variation of fitness was significantly related to protein expression, and if not significant, it was unrelated. For the interaction effect of hybridization and protein expression, wild soybeans were divided into one group, and F_2_-positive homozygotes and heterozygotes were divided into another group; if the difference was significant, it was significantly related to the interaction effect, but if not significant, it was unrelated. All the above statistical analyses were carried out using IBM SPSS Statistics 20 software.

## Results

### Analysis of *EPSPS* Expression in Hybrid Progeny of Wild and Transgenic Soybeans

#### Detection of Foreign Gene Genotypes

In 2019, the Ct values of the exogenous and endogenous genes for lectin of the F_2_ generation were determined using real-time fluorescence quantitative PCR. According to the Ct values, 27 negative and 93 positive samples were identified. The *EPSPS* protein test strip was used to detect expression in soybean samples, and the results of the test strip were consistent with the results of the quantitative PCR test (Supplementary Table S1). The copy numbers of the foreign gene and lectin gene in the F_2_ generation were quantified by digital PCR twice for each sample. The ratio of *EPSPS* gene copy number to lectin gene copy number in each sample was calculated according to the measured copy number, and the homozygosity of the sample was determined. A total of 25 non-transgenic negative plants, 56 heterozygous plants, and 39 positive homozygous plants were detected, with a quantitative ratio of 0.83:1.87:1.3, in accordance with the Mendelian genetic law (X^2^ = 3.80, df = 2, 0.150, *p* = 0.150, on a Chi-square test; Supplementary Table S2).

#### Detection of *EPSPS* Protein Expression

In 2018, the expression of *EPSPS* protein in hybrid F_1_ soybeans was significantly lower than that in transgenic soybeans (*p* < 0.0001). The results in 2019 showed that the expression of *EPSPS* protein in F_2_ homozygous positive plants was slightly higher than that in heterozygous positive plants and hybrid F_1_ soybeans, but not at significantly. The expression of *EPSPS* protein in F_2_ homozygous and heterozygous soybeans was significantly lower than that in transgenic soybeans (*p* < 0.0001; Table 3). The above results showed that the foreign protein could be expressed normally in the hybrid offspring of transgenic and wild soybeans, but the level of protein expression was significantly lower than that of transgenic soybeans.

**Table 3:** Expression of *EPSPS* protein in apical leaves of transgenic soybean and hybrid progeny at 3–4 leaf stage (μg/g).

Year	Soybean materials	Expression of *EPSPS* protein
2018	GM soybean	227.9 ± 37.3a
	F_1_	141.6 ± 12.7b
2019	GM soybean	232.5 ± 35.9a
	F_2_-Positive homozygote	165.4 ± 17.8b
	F_2_-Heterozygote	156.8 ± 20.0b

*The data are expressed as average ± standard deviation, and the values marked with the same letters are not significantly different (p > 0.05)*.

### Growth Traits

#### Emergence Rate

**Figure 1:**
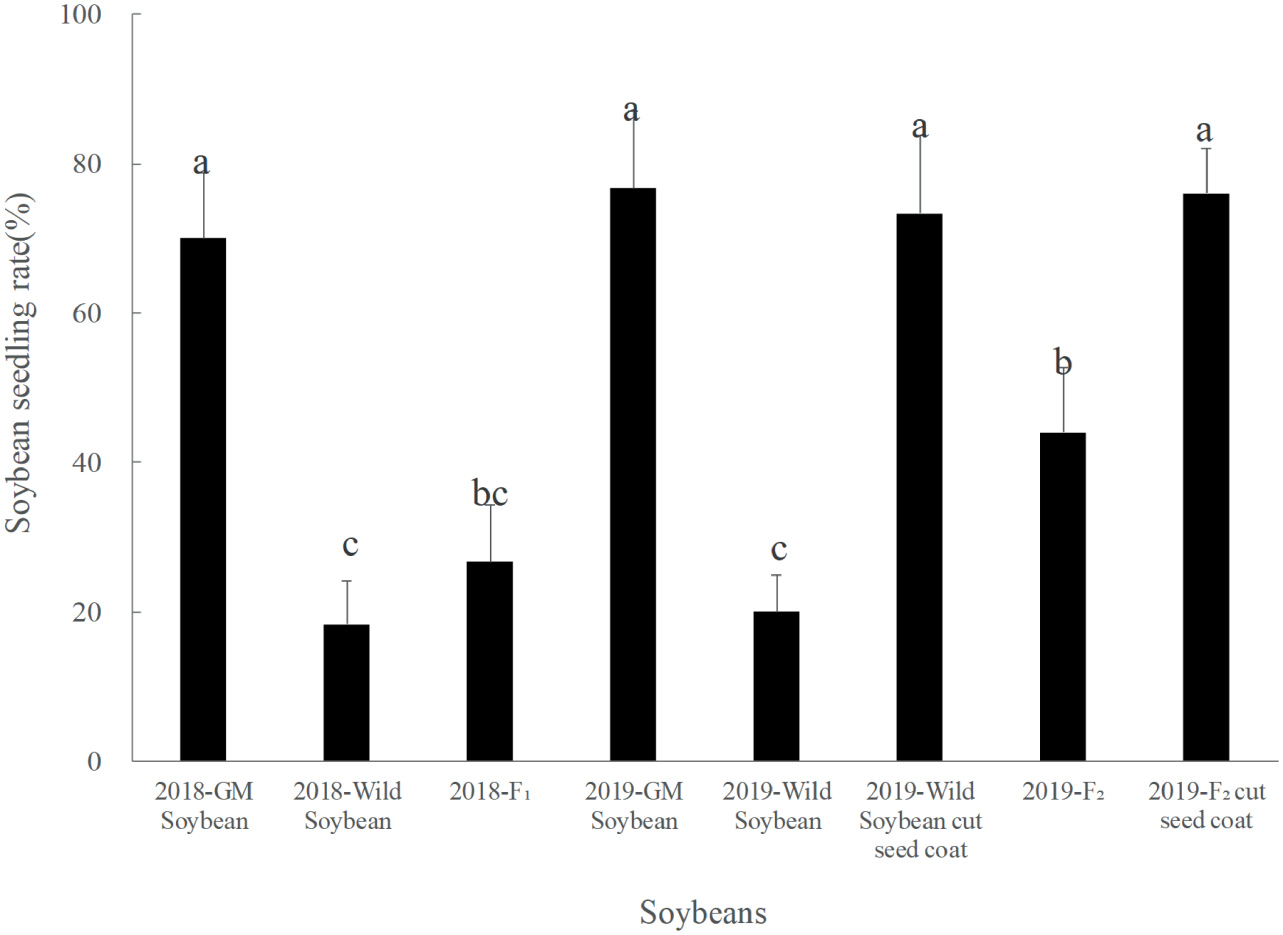
Emergence rate (%) of transgenic, wild soybeans, and hybrid progeny. The letters indicate that there is no significant difference in seedling emergence rate among soybean materials (*p* > 0.05).

In 2018, the seed emergence rate of transgenic soybeans was the highest, followed by the hybrid F_1_ generation, and then the wild soybean, which showed the lowest rate (Figure 1). The emergence rate of hybrid F_1_ was significantly lower than that of transgenic soybeans (p < 0.0001), but not significantly different from that of wild soybeans (*p* < 0.941). The emergence rate of wild soybeans was significantly lower than that of transgenic soybeans (*p* < 0.0001). Although the emergence ability of the hybrid F_1_ generation is low, some seeds can germinate normally, especially if the basic conditions for population continuation are present.

The emergence rate of the F_2_ generation in 2019 was significantly lower than that of transgenic soybeans and significantly higher than that of wild soybeans. The emergence rate of the F_2_ generation was higher than that of the hybrid F_1_ generation, but the difference was not significant (*p* > 0.05). The germination rate of wild soybeans and F_2_ seeds was significantly higher than that before peeling, and there was no significant difference between the wild and transgenic soybeans (*p* > 0.05). Hence, F_2_ soybean seeds could germinate normally, and foreign genes were capable of long-term existence in wild soybeans.

#### Growth Period

**Figure 2:**
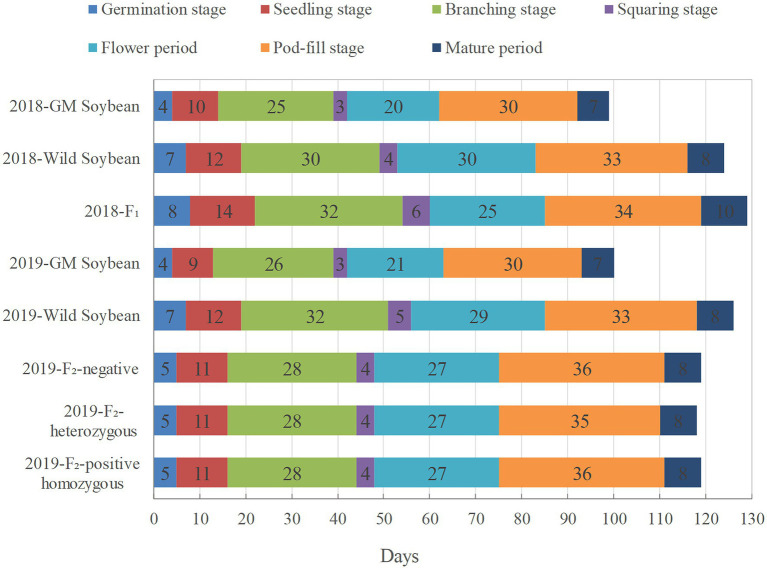
Growth periods of genetically modified, wild soybeans, and hybrid offspring (days).

In 2018, hybrid F_1_ soybeans had the longest growth period (129 days), followed by wild soybeans (124 days), and transgenic soybeans (98 days; Figure 2). Among them, the flowering period of hybrid F_1_ soybeans overlapped with that of wild soybeans for 23 days, indicating that the foreign genes of hybrid progeny had the objective conditions to continue to spread to wild soybeans, and there was a 2-day overlap with the flowering period of transgenic soybeans. This shows that the foreign gene possessed the ability to further spread to the hybrid F_1_ generation.

During the growth period in 2019, the growth periods of homozygous positive plants, heterozygous positive plants, and negative plants of F_2_ soybeans were 119, 118, and 119 days, respectively, falling between those of wild soybeans (126 days) and transgenic soybeans (100 days; Figure 2). The flowering periods of the three genotypes of soybean in the F_2_ generation were almost the same, overlapping with that of wild soybean for 21 days. It is inferred that if the genetically modified soybean pollen drifts to the wild soybeans, the hybrid F_2_ soybean still has the possibility of spreading the *EPSPS* gene to other wild soybean individuals in the natural population.

#### Aboveground Biomass

**Figure 3:**
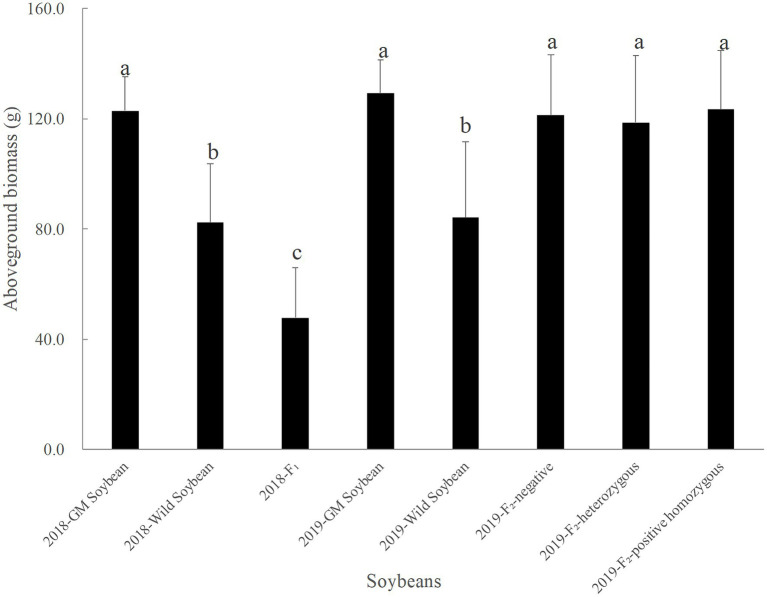
Aboveground biomass of transgenic, wild soybeans, and hybrid progeny (g).

Figure 3 shows that in 2018, the hybrid F_1_ generation had the smallest average aboveground biomass, followed by wild and transgenic soybeans. The average aboveground biomass of the hybrid F_1_ was significantly lower than that of transgenic (*p* < 0.0001) and wild soybeans (*p* = 0.0001), and the average aboveground biomass of wild soybeans was significantly lower than that of transgenic soybeans (*p* < 0.0001). The results showed that the vegetative growth ability of the hybrid F_1_ was significantly lower than that of its two parents, showing a significant cost to the plant’s health.

In 2019, transgenic soybeans had the highest average aboveground biomass, followed by the F_2_ generation soybeans, and then transgenic soybeans, which had the lowest value. There was no significant difference in the average aboveground biomass values among F_2_ soybean-negative plants, homozygous positive plants, and heterozygous positive plants (*p* > 0.05). The average aboveground biomass of wild soybeans was significantly lower than that of transgenic soybeans (*p* < 0.001) and F_2_ soybean populations (*p* < 0.0001). The results of the correlation analysis showed that the change in aboveground biomass was significantly correlated with hybridization (*p* < 0.0001) and the interaction between hybridization and protein expression (*p* < 0.05), but non-significantly correlated with protein expression (*p* = 0.796). Compared to the F_1_ generation, the average aboveground biomass of the F_2_ generation soybean increased significantly (*p* < 0.0001), indicating that the vegetative growth ability of the F_2_ generation soybean was significantly higher than that of its parent, F_1_ generation soybean. Compared to wild soybeans, the F_2_ generation soybeans also showed a significant fitness advantage, which was beneficial for further diffusion of foreign genes in the wild soybean population.

### Reproductive Index

#### Pollen Germination Rate

**Figure 4:**
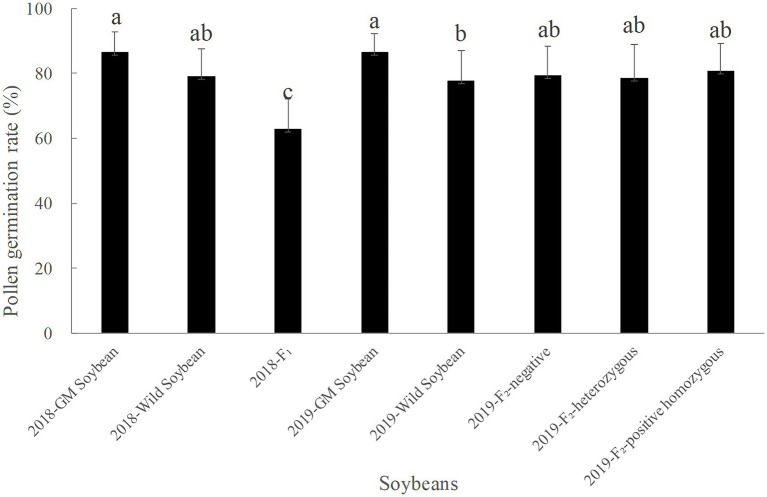
Pollen germination rate (%) of transgenic, wild soybeans, and hybrid offspring.

Figure 4 shows that the average pollen germination rate in soybeans in 2018 was the highest for transgenic, followed by wild soybeans, and then F_1_ generation, which had the lowest rate. The average pollen germination rate of hybrid F_1_ was significantly lower than that of transgenic (*p* < 0.0001) and wild soybeans (*p* = 0.0001). The average pollen germination rate of wild soybeans was significantly lower than that of transgenic (*p* = 0.046).

In 2019, the average pollen germination rate was the highest for transgenic, followed by the F_2_ generation, and then wild soybeans, which had the lowest value. There was no significant difference in the pollen germination rate among the F_2_ generation negative plants, homozygous positive plants, and heterozygous positive plants (*p* > 0.05). The pollen germination rate of wild was significantly lower than that of transgenic soybeans (*p* < 0.05).

#### Number of Pods per Plant

**Figure 5:**
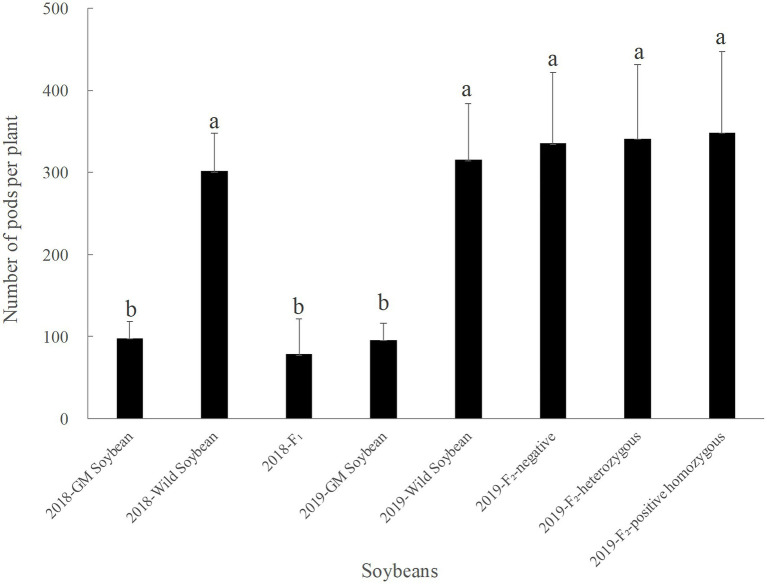
Pods per plant of genetically modified, wild soybeans, and hybrid progenies.

In 2018, the average pod number per plant was the highest for wild, followed by transgenic soybeans, and then the F_1_ generation, which had the lowest number (Figure 5). There was no significant difference in the numbers between hybrid F_1_ and transgenic soybeans (*p* = 0.258), but both were significantly lower than the number of pods for wild soybeans (*p* < 0.0001).

In 2019, the number of pods per plant was the lowest for transgenic, followed by wild soybeans; the number for the F_2_ generation was the highest (Figure 5). There was no significant difference in the number of pods per plant among F_2_ soybean-negative plants, homozygous positive plants, heterozygous positive plants, and wild soybeans (*p* > 0.005). However, the number for F_2_ generation was significantly higher than that for transgenic soybeans (*p* < 0.0001). The results showed that the number of pods per plant for hybrid F_2_ was greater than that for wild soybeans, although there was no significant difference, indicating a certain advantage of fitness that was beneficial for the further diffusion of foreign genes.

#### Full Grains per Pod

Table 4 shows that the average seed number per full pod in 2018 was the highest for genetically modified, followed by wild, and the hybrid F_1_ generation soybeans, which showed the lowest number. The average number of full seeds per pod for the hybrid F_1_ generation was significantly lower than that for transgenic soybeans (*p* < 0.0001) and wild soybeans (*p* = 0.037), and there was no significant difference in the number of full seeds per pod between wild and transgenic soybeans (*p* = 0.206), indicating that the hybrid F_1_ generation had a significant fitness cost compared to its parents.

**Table 4:** Number of full grains per pod for transgenic, wild soybeans, and hybrid offspring.

Year	Soybean	Number of grains per plant
2018	GM soybean	2.30 ± 0.23a
	Wild soybean	2.09 ± 0.15a
	F_1_	1.82 ± 0.24b
2019	GM soybean	2.30 ± 0.16a
	Wild soybean	2.10 ± 0.15a
	F_2_-Negative	2.11 ± 0.14a
	F_2_-Heterozygote	2.12 ± 0.23a
	F_2_-Positive homozygote	2.10 ± 0.16a

In 2019, the average number of full grains per pod was the highest for transgenic, followed by the F_2_ generation and wild soybeans. There was no significant difference in the average number of full seeds per pod among the F_2_ generation soybean-negative plants, homozygous positive plants, and hybrid positive plants (*p* > 0.05), and no significant difference between the F_2_ generation and transgenic soybeans (*p* = 0.260). Compared to the F_1_ hybrid generation, the number of full grains per pod in F_2_ soybeans increased significantly (*p* < 0.05), showing a fitness advantage, which was conducive to the further spread of foreign genes.

#### Number of Full Grains per Plant

**Figure 6:**
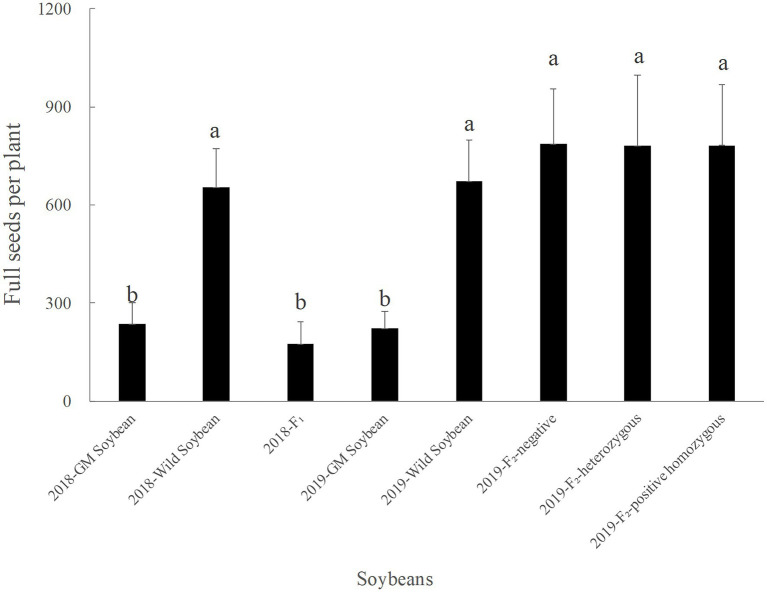
Number of full grains per plant (grains) for genetically modified, wild soybeans, and hybrid progenies.

The results in Figure 6 show that the average number of full grains per plant in 2018 were the highest for wild, followed by transgenic, and hybrid F_1_ soybeans. The average number of full grains per plant for hybrid F_1_ was not significantly different from that for transgenic soybeans (*p* = 0.974), but significantly lower than that for wild soybeans (*p* < 0.0001), indicating that the cost of fitness of the hybrid F_1_ generation was significant compared with that of wild soybeans.

In 2019, the average number of full grains per plant was the lowest for transgenic, followed by wild and F_2_ generation soybeans. There was no significant difference in the number of filled grains per plant between the three phenotypes of the F_2_ generation and wild soybeans (*p* > 0.05), but the values were significantly higher than that for transgenic soybeans (*p* < 0.0001). The number of full grains per plant for the F_2_ generation was significantly higher than that for the hybrid F_1_ generation (*p* < 0.0001). The results showed that the seed production ability of the F_2_ generation was significantly higher than that of F_1_ generation and wild soybeans, which was conducive to the long-term retention of foreign genes in the hybrid progeny.

#### Hundred-Grain Weight

The results in Table 5 show that in 2018, the average 100-grain weight was the highest for genetically modified, followed by wild and hybrid F_1_ soybeans. There was no significant difference in the average 100-seed weight between the hybrid F_1_ and wild soybeans (*p* = 1.000), but both values were significantly lower than those for transgenic soybeans (*p* < 0.0001).

**Table 5:** Hundred-seed weight of genetically modified, wild soybeans, and hybrid progeny (g).

Year	Soybean	Hundred-seed weight (g)
2018	GM soybean	15.7 ± 1.26a
	Wild soybean	2.05 ± 0.10c
	F_1_	2.20 ± 0.17bc
2019	GM soybean	15.1 ± 1.19a
	Wild soybean	2.03 ± 0.13c
	F_2_-Negative	2.98 ± 0.13b
	F_2_-Heterozygote	2.96 ± 0.14b
	F_2_-Positive homozygote	2.95 ± 0.09b

In 2019, the average 100-seed weight was the highest for transgenic, followed by the F_2_ generation and wild soybeans. The 100-seed weight of the F_2_ generation negative plants, homozygous positive plants, and hybrid positive plants showed no significant difference (*p* > 0.05), but the 100-seed weight of the F_2_ generation was significantly higher than that of wild soybeans (0.01 < *p* < 0.05) and significantly lower than that of transgenic soybeans (*p* < 0.0001). Compared to the F_1_ hybrid generation, the 100-seed weight of the F_2_ soybeans showed no obvious change (*p* > 0.05). Correlation analysis showed that the change in 100-seed weight was significantly correlated with hybridization (*p* < 0.05), but not with protein expression (*p* > 0.05). The results showed that the seed weight of hybrid progeny was significantly higher than that of wild soybeans, showing the benefit of fitness that was beneficial to the diffusion of foreign genes.

### Correlation and Interaction Between Fitness Index, Hybridization, and Protein Expression

Compared to the wild parent soybeans, there was no significant difference in seedling emergence rate, pollen germination rate, full seeds per pod, pods per plant, and full seeds per plant between the F_2_ generation and wild soybean, but the aboveground biomass and 100-seed weight of the former were significantly higher than those of the latter. The correlation between the change in fitness index, hybridization, and protein expression, and the interaction between them are shown in Table 6. The fitness indices with significant differences were not related to protein expression but were significantly related to hybridization and the interaction between it and protein expression.

**Table 6:** Correlation and interaction between fitness index, hybridization, and protein expression.

Fitness index	Correlation with hybridization	Correlation with protein expression	Correlation with interaction between hybridization and protein expression
Emergence rate	N	N	N
Aboveground biomass	^***^	N	^***^
Pollen germination rate	N	N	N
Number of pods per plant	N	N	N
Number of full grains per pod	N	N	N
Number of full grains per plant	N	N	N
100-grain weight	^*^	N	^*^

“N” indicates that the fitness index has no significant difference or is not related to hybridization or protein expression (p > 0.05). “^*^” indicates that the change of fitness index is significantly correlated with hybridization or protein expression (0.01 < p < 0.05). “^***^” indicates that the change of fitness index is significantly related to hybridization or protein expression (p < 0.0001).

### Relative Fitness

In 2018, compared to wild soybeans, the vegetative reproductive indices of hybrid F_1_ showed a significant cost to the health of the plants. In 2019, compared to wild soybeans, F_2_ soybeans showed a certain fitness advantage with respect to aboveground biomass and 100-grain weight, and there was no significant difference in the seedling emergence rate, pollen germination rate, pods per plant, and full grains per plant. There was no significant difference in the nutrition and reproductive indices among the F_2_ soybean-negative plants, homozygous positive plants, and heterozygous positive plants, while the relative fitness of the F_2_ was significantly higher than that of hybrid F_1_ soybeans with wild soybeans as a common control (Table 7). The results showed that, compared with wild soybeans, although the F_1_ generation of hybrid progeny had a significant fitness cost, the fitness returned to the advantage in the F_2_ generation. Overall, foreign genes can be retained in hybrid offspring and spread with the reproduction of hybrid offspring, which can result in certain ecological impacts.

**Table 7:** Changes in fitness of vegetative reproductive indices of hybrid F_1_ and F_2_ generation compared to wild soybeans.

Materials Index	2018		2019			
	Wild soybean	F_1_	Wild soybean	F_2_-negative plants	F_2_-hybrid plants	F_2_-positive homozygous plants
Emergence rate	1.00 ± 0.19a	0.40 ± 0.11b	1.00 ± 0.14a	1.13 ± 0.10a		
Aboveground biomass	1.00 ± 0.26a	0.58 ± 0.22b	1.00 ± 0.33b	1.44 ± 0.26a	1.41 ± 0.29a	1.47 ± 0.25a
Pollen germination rate	1.00 ± 0.11a	0.80 ± 0.11b	1.00 ± 0.12a	1.02 ± 0.11a	1.01 ± 0.13a	1.04 ± 0.11a
Number of pods per plant	1.00 ± 0.15a	0.26 ± 0.14b	1.00 ± 0.22a	1.06 ± 0.27a	1.08 ± 0.29a	1.10 ± 0.31a
Number of full grains per plant	1.00 ± 0.18a	0.27 ± 0.10b	1.00 ± 0.19a	1.17 ± 0.25a	1.16 ± 0.32a	1.16 ± 0.27a
100-seed weight	1.00 ± 0.05a	1.07 ± 0.08a	1.00 ± 0.06b	1.47 ± 0.06a	1.46 ± 0.07a	1.45 ± 0.04a
Total fitness	1.00	0.60	1.00	1.23	1.22	1.24

*Peers are marked with the same letters in the same year, indicating that there is no significant difference in the fitness indicators (p > 0.05)*.

## Discussion

When the flowering periods of crops overlap with those of their wild species, and the ecological suitability of the hybrid progenies in the farmland ecosystem is consistent with that of at least the wild parents, foreign genes can exist in wild species for a long time (Sahoo et al., 2010), whereas the existence of foreign genes in wild species can be reduced when the gametes are physically separated in space or time by cross-incompatibility or by decreased fitness of the F_1_ progeny (Ellstrand and Hoffman, 1990; Spencer and Snow, 2001; Lee and Natesan, 2006). Previous studies have shown that cultivated soybeans can transfer their genes to wild soybeans through pollen transmission, resulting in wild soybeans containing some genes of cultivated soybeans (Nakayama and Yamaguchi, 2002; Kuroda et al., 2006; Wang and Li, 2013a,b). Therefore, to protect the diversity of wild soybeans, it is necessary to examine the fitness and foreign protein expression of the hybrid offspring of transgenic and wild soybeans, as done in this study.

### Analysis of *EPSPS* Gene Expression in Progeny of Transgenic and Wild Soybeans

In this study, the top leaf samples of soybean plants were collected at the 3–4-leaf stage to detect the expression level of *EPSPS* foreign protein in transgenic soybeans and hybrid progeny. Chinnadurai et al (2018) collected different soybean tissues from 74 field experiments under different environments and extensively tested the expression level of *CP4-EPSPS*. The expression levels of *CP4-EPSPS* in the leaves collected at four different stages of plant development (V3-V5, V4-V9, R1-R3, and R3-R6) were not significantly different, but were significantly higher than the levels in the stems, seeds, and roots. Therefore, the level of *EPSPS* expression measured in this experiment can represent the true level of *EPSPS* expression in transgenic and hybrid soybeans.

The copy number of gene can affect its expression level (Stranger et al., 2007). According to the results of this study, *EPSPS* foreign protein could be expressed normally in the hybrid offspring of transgenic and wild soybeans, but the protein expression level was significantly lower than that in transgenic soybeans. Items containing two *cp4 esps* loci had higher protein expression and tended to express about twice as much as those containing a single *cp4 esps* locus in all soybean tissue types (Chinnadurai et al., 2018). In this study, the expression of *EPSPS* protein in F_2_ homozygous positive plants was slightly higher than that in heterozygous positive plants and hybrid F_1_ soybeans, but there was no significant difference between them (*p* > 0.05). It has been confirmed that foreign genes can drift from genetically modified into wild soybeans and can be passed on through self-crossing of hybrid offspring (Yook et al., 2021). We noticed that the expression of foreign proteins in F_2_ homozygous positive plants was higher than that in heterozygous plants, but there was no significant difference, which may be due to the presence of a single harvest of F_1_ seeds in this experiment and because the planted F_2_ plants came from different parents with different *EPSPS* expression levels. The relationship between protein expression and homozygous and heterozygous phenotypes can be determined by detecting the protein expression in homozygous positive and heterozygous plants of the same parent.

The results of previous studies showed that the foreign protein could be expressed normally in the hybrid offspring of transgenic crops crossed with weeds or wild species (Pandolfo et al., 2018), which is consistent with the results of this study. The expression of foreign proteins can confer hybrid progeny with the same or similar resistance as transgenic crops, resulting in unpredictable environmental risks (Dale, 1992; Snow et al., 1999; Stewart Jr et al., 2003; Lu, 2013; Huang et al., 2019). In Argentina, transgenic rapeseed with the *CP4-EPSPS* gene passes it on to the wild-type turnip, resulting in glyphosate tolerance and control problems (Pandolfo et al., 2018). Therefore, the change in fitness of wild soybeans after obtaining the *EPSPS* gene was evaluated in this experiment, to clarify the ecological risk of *EPSPS* drifting to wild soybeans.

### Analysis of Fitness of Hybrid Progeny of Transgenic and Wild Soybeans

Dissemination of transgenic plants through pollen is the most common method of gene flow, where transgenic crops are hybridized with their sexually compatible wild relatives through pollen transmission, with the foreign genes thus escaping to the natural environment (Liu et al., 2020). Therefore, the overlap of flowering is one of the necessary conditions for gene flow. Herbicides were not used throughout the growth process of the soybeans to simulate the growth of hybrid offspring of transgenic and wild soybeans in the natural ecosystem. At the same planting time, the florescence of hybrid F_1_ and wild soybeans overlapped for 23 days, and the flowering period of F_2_ and wild overlapped for 21 days. The results showed that the foreign genes in the hybrid progeny possessed the ability to further spread to the wild soybean population. It is necessary to further study the fitness of hybrid progeny and wild soybean backcross progeny. In this experiment, the values of most of the fitness indices of the hybrid F_1_ generation were significantly lower than those of the parent wild soybean, which may be due to inflorescence damage and poor seed development in the process of hybridization. Therefore, the change in fitness of the F_2_ generation was explored in this study. There was no significant difference in vegetative and reproductive growth between F_2_-negative and F_2_-positive heterozygote and homozygous plants, indicating that the insertion of the *EPSPS* gene does not affect the growth and development of wild soybeans. In the absence of herbicide selection pressure, the aboveground biomass and 100-seed weight of F_2_ soybeans were significantly higher than those of wild soybeans, and there was no significant difference in the emergence rate, pollen germination rate, pods per plant, full seeds per pod, and full seeds per plant between F_2_ and wild soybeans. The results showed that the hybrid progeny (F_2_) of transgenic and wild soybeans had no fitness cost in terms of nutrition and reproductive indices, which was beneficial to the diffusion of foreign genes in the wild soybean population. In contrast to the results of this study, Kuroda et al (2013) suggested that the hybrid progeny of cultivated and wild soybeans contain some artificial domestication genes, so the fitness of hybrid progeny of cultivated and wild soybeans may be lower than that of wild soybeans. When transgenic soybeans with the *EPSPS* gene (developed by Nanjing Agricultural University) were crossed with wild soybeans from 10 different regions, the seed germination percentage, plant height, biomass, pods per plant, and 100-seed weight of F_1_ were lower than those of wild soybeans, indicating that the gene transfer was not conducive to the survival of hybrid offspring in natural environments (Liu et al., 2021).

Further, the results of three reports on the fitness of hybrid progeny of *EPSPS* transgenic and wild soybeans are similar to those in this study. Guan et al (2015) found that the pod setting rate of *EPSPS* transgenic soybean AG5601 and Beijing wild soybean hybrids was very low, and there was no significant difference in plant growth between F_2_ hybrids with the *EPSPS* gene and those without it. Herbicide-resistance genes did not seem to adversely affect the growth of introduced wild soybeans, indicating that the escaped *EPSPS* gene may persist in environments where herbicides are not used. Kan et al (2015) studied the fitness of four wild soybean materials and hybrid progeny of glyphosate-resistant *EPSPS-*transgenic soybean RR under net room-temperature conditions. In the absence of glyphosate selection pressure, the relative fitness of hybrid progeny with respect to some reproductive traits, such as pod setting rate and 100-seed weight, was higher than that of the female parents, but there was no significant difference in other fitness indices. Neither was there a significant difference in the values of the fitness indices between F_2_-negative plants and F_2_-positive plants. Yook et al (2021) assessed the potential weed risk of hybrid progeny caused by gene flow from glyphosate-resistant (GR) soybeans to their wild relatives in South Korea through a 2-year field study. Hybrid F_1_ and F_2_ showed intermediate characteristics of the parent soybean in the vegetative and reproductive growth stages. The canopy height and stem length of hybrid progeny were similar to those of wild soybeans, and the number of flowers, pods, and seeds per plant were similar to those of wild soybeans, but significantly higher than those of transgenic soybeans. Owing to the high dormancy of seeds, the seed lifespan of the F_2_ generation was also modest, but significantly higher than that of transgenic soybeans. The results showed that, due to pollen-mediated gene flow and high fitness of hybrid offspring, genetically modified genes may be dispersed into wild populations and persist in the local agro-ecosystem. The above results showed that, compared with wild soybeans without glyphosate, the fitness of those expressing *EPSPS* did not change significantly and even showed significant improvement in some fitness indices. Therefore, the offspring of transgenic and wild soybeans may be more invasive than their wild soybean parents, resulting in the spread of the foreign *EPSPS* gene in the wild soybean population.

Using the above examples, a clear conclusion about the fitness of hybrid offspring of genetically modified and wild soybeans cannot be reached, and further systematic and in-depth research should be carried out according to the principle of “case analysis.”

### Ecological Risk Analysis of Progeny of Transgenic and Wild Soybeans

When there is a high level of gene communication between crops and wild species, their hybridization can threaten the wild population in two ways: First, by demographic swamping caused by the decrease in the population growth rate due to the decrease in the fitness level of the hybrid offspring, and second, by genetic swamping caused by the substitution of pure wild genotypes by the hybrid progeny with medium-to-high fitness over time (Wolf et al., 2001; Todesco et al., 2016). Some studies have shown that the suitability of hybrid offspring of crops and wild species is lower than that of their wild parents, for example, in sunflower (Gutierrez et al., 2011). However, in many other studies, the suitability of hybrids has not declined, such as of sorghum (Schmidt et al., 2018). Some hybrids even showed stronger adaptability than their parent species, such as for lettuce (Hooftman et al., 2007) and radish (Hovick et al., 2012). In this experiment, some fitness indices of the hybrid progeny of transgenic and wild soybeans were higher than those of wild soybeans, which may threaten their diversity. Therefore, in this study, we showed that, to assess the threat of hybridization between modified crops and wild species to the diversity of wild species, it is necessary to estimate not only the frequency of gene flow, but also the relative fitness of hybrid offspring (Feurtey et al., 2017).

## Data Availability Statement

The original contributions presented in the study are included in the article/Supplementary Material, further inquiries can be directed to the corresponding authors.

## Author Contributions

LL carried out the experiments, analyzed the data, and wrote the manuscript. LZ helped carry out the experiments. JF, WS, ZF, YD, and RJ helped analyze the data. JL and BL designed and helped to calibrate the manuscript. All authors contributed to the article and approved the submitted version.

## Funding

This research was supported by the National Natural Science Foundation of China (32171656), the National Special Transgenic Project of China (2016ZX08012005), the Basic Scientific Research Program of National Nonprofit Research Institutes (GYZX200103), and the Natural Science Foundation of Jiangsu Province (grants no BK 20200157).

## Conflict of Interest

The authors declare that the research was conducted in the absence of any commercial or financial relationships that could be construed as a potential conflict of interest.

## Publisher’s Note

All claims expressed in this article are solely those of the authors and do not necessarily represent those of their affiliated organizations, or those of the publisher, the editors and the reviewers. Any product that may be evaluated in this article, or claim that may be made by its manufacturer, is not guaranteed or endorsed by the publisher.
