# Supplementary material for: Fitness and Ecological Risk of Hybrid Progenies of Wild and Herbicide-Tolerant Soybeans With EPSPS Gene
Source: Front Plant Sci. 2022 Jun 9;13:922215. doi: 10.3389/fpls.2022.922215 (PMC9224928; doi:10.3389/fpls.2022.922215)
Supplement: Supplementary file 1 [file Data_Sheet_1.docx]

**Supporting Information**

**Fitness and ecological risk of hybrid progenies of wild and herbicide-tolerant soybeans with *EPSPS* gene**

**Laipan Liu^1,2^, Li Zhang^1^, Jianmei Fu^1^, Wenjing Shen^1^, Zhixiang Fang^1^, Ying Dai^1^, Ruizong Jia^3^, Biao Liu^1,2*^, Jingang Liang^4^**

^1^Key Laboratory on Biosafety of Nanjing Institute of Environmental Sciences, Ministry of Ecology and Environment, Nanjing, China

^2^State Environmental Protection Scientific Observation and Research Station for Ecology and Environment of Wuyi Mountains, Nanjing Institute of Environmental Sciences, Ministry of Ecology and Environment, Nanjing, China

^3^Hainan Key Laboratory for Biosafety Monitoring and Molecular Breeding in Off-Season Reproduction Regions, Sanya, China

^4^Development Center of Science and Technology, Ministry of Agriculture and Rural Affairs, Beijing 100176

*** Correspondence:**Biao Liu
[liubiao@nies.org](mailto:liubiao@nies.org)

Jingang Liang
liangjingang@agri.gov.cn

**Keywords: Glyphosate-tolerant soybeans, Gene flow, Hybrid progeny, Relative fitness, Ecological risk**

**Supplementary Tables**

**Table S1** Quantitative PCR determination of soybean leaf samples and results of epsps protein test strip

| Material number | Ct value of foreign gene | Ct value of Lectin | ∆ct | Result determination | Test strip |
| --- | --- | --- | --- | --- | --- |
|  |  |  |  |  | Negative (-) / Positive(+) |
| F_2_-1 | 26.25 | 25.68 | 0.57 | Positive | + |
| F_2_-2 | 27.86 | 26.8 | 1.06 | Positive | + |
| F_2_-3 | 26.04 | 26.92 | -0.88 | Positive | + |
| F_2_-4 | Undetermined | 27.08 |  | Negative | - |
| F_2_-5 | 26.21 | 25.53 | 0.68 | Positive | + |
| F_2_-6 | Undetermined | 25.75 |  | Negative | - |
| F_2_-7 | 29.31 | 29.73 | -0.42 | Positive | + |
| F_2_-8 | 28.1 | 27.98 | 0.12 | Positive | + |
| F_2_-9 | 29.44 | 29.67 | -0.23 | Positive | + |
| F_2_-10 | 28.73 | 29.73 | -1.01 | Positive | + |
| F_2_-11 | 27.71 | 29.02 | -1.32 | Positive | + |
| F_2_-12 | 26.28 | 27.34 | -1.07 | Positive | + |
| F_2_-13 | Undetermined | 27.91 |  | Negative | - |
| F_2_-14 | 27.51 | 27.78 | -0.26 | Positive | + |
| F_2_-15 | 26.82 | 26.98 | -0.16 | Positive | + |
| F_2_-16 | 26.65 | 26.56 | 0.09 | Positive | + |
| F_2_-17 | Undetermined | 29.47 |  | Negative | - |
| F_2_-18 | 27.11 | 27.02 | 0.09 | Positive | + |
| F_2_-19 | Undetermined | 27.6 |  | Negative | - |
| F_2_-20 | 26.46 | 26.62 | -0.16 | Positive | + |
| F_2_-21 | 25.86 | 25.81 | 0.06 | Positive | + |
| F_2_-22 | Undetermined | 19.83 |  | Negative | - |
| F_2_-23 | 27 | 27.3 | -0.3 | Positive | + |
| F_2_-24 | 27.46 | 27.83 | -0.37 | Positive | + |
| F_2_-25 | 26.88 | 27.01 | -0.13 | Positive | + |
| F_2_-26 | 25.88 | 26.16 | -0.29 | Positive | + |
| F_2_-27 | 26.49 | 26.73 | -0.24 | Positive | + |
| F_2_-28 | 27.79 | 28.7 | -0.91 | Positive | + |
| F_2_-29 | Undetermined | 27.75 |  | Negative | - |
| F_2_-30 | Undetermined | 30.43 |  | Negative | - |
| F_2_-31 | 28.86 | 28.6 | 0.25 | Positive | + |
| F_2_-32 | 24.54 | 25.14 | -0.6 | Positive | + |
| F_2_-33 | 24.68 | 25.03 | -0.35 | Positive | + |
| F_2_-34 | 25.51 | 24.97 | 0.54 | Positive | + |
| F_2_-35 | Undetermined | 31.61 |  | Negative | - |
| F_2_-36 | 26.17 | 25.75 | 0.42 | Positive | + |
| F_2_-37 | 25.11 | 25.25 | -0.14 | Positive | + |
| F_2_-38 | Undetermined | 27.44 |  | Negative | - |
| F_2_-39 | 24.89 | 25.86 | -0.98 | Positive | + |
| F_2_-40 | 24.44 | 25.33 | -0.89 | Positive | + |
| F_2_-41 | 25.66 | 26.83 | -1.17 | Positive | + |
| F_2_-42 | 27.48 | 28.47 | -0.99 | Positive | + |
| F_2_-43 | 25.84 | 25.03 | 0.81 | Positive | + |
| F_2_-44 | 26.86 | 25.95 | 0.91 | Positive | + |
| F_2_-45 | 25.53 | 25.61 | -0.08 | Positive | + |
| F_2_-46 | Undetermined | 26.77 |  | Negative | - |
| F_2_-47 | 24.97 | 24.79 | 0.18 | Positive | + |
| F_2_-48 | 26.9 | 27.04 | -0.14 | Positive | + |
| F_2_-49 | Undetermined | 27.63 |  | Negative | - |
| F_2_-50 | 26.46 | 26.95 | -0.49 | Positive | + |
| F_2_-51 | Undetermined | 27.28 |  | Negative | - |
| F_2_-52 | 23.96 | 24.67 | -0.71 | Positive | + |
| F_2_-53 | 24.21 | 23.54 | 0.67 | Positive | + |
| F_2_-54 | 24.74 | 25.16 | -0.42 | Positive | + |
| F_2_-55 | 23.93 | 23.08 | 0.85 | Positive | + |
| F_2_-56 | 25.28 | 25.42 | -0.14 | Positive | + |
| F_2_-57 | Undetermined | 29.53 |  | Negative | - |
| F_2_-58 | 21.76 | 22.5 | -0.74 | Positive | + |
| F_2_-59 | 23.57 | 24.46 | -0.89 | Positive | + |
| F_2_-60 | 23.98 | 24.89 | -0.91 | Positive | + |
| F_2_-61 | Undetermined | 29.26 |  | Negative | - |
| F_2_-62 | 22.51 | 22.73 | -0.22 | Positive | + |
| F_2_-63 | 25.27 | 25.92 | -0.65 | Positive | + |
| F_2_-64 | 25.95 | 25.34 | 0.61 | Positive | + |
| F_2_-65 | 25.55 | 24.92 | 0.63 | Positive | + |
| F_2_-66 | 24.45 | 23.69 | 0.76 | Positive | + |
| F_2_-67 | Undetermined | 25.96 |  | Negative | - |
| F_2_-68 | 25.22 | 25.99 | 0.77 | Positive | + |
| F_2_-69 | 26.36 | 27.05 | -0.69 | Positive | + |
| F_2_-70 | 21.95 | 21.43 | 0.52 | Positive | + |
| F_2_-71 | 20.48 | 20.69 | -0.21 | Positive | + |
| F_2_-72 | 26.5 | 25.55 | 0.95 | Positive | + |
| F_2_-73 | 26.5 | 26.55 | -0.05 | Positive | + |
| F_2_-74 | Undetermined | 26.7 |  | Negative | - |
| F_2_-75 | Undetermined | 26.16 |  | Negative | - |
| F_2_-76 | 26.65 | 25.92 | 0.73 | Positive | + |
| F_2_-77 | 25.85 | 25 | 0.85 | Positive | + |
| F_2_-78 | 25.25 | 25.72 | 0.47 | Positive | + |
| F_2_-79 | Undetermined | 24.79 |  | Negative | - |
| F_2_-80 | 25.53 | 26.61 | -1.08 | Positive | + |
| F_2_-81 | 22.11 | 21.3 | 0.81 | Positive | + |
| F_2_-82 | 25.43 | 24.89 | 0.54 | Positive | + |
| F_2_-83 | 23.88 | 23.45 | 0.43 | Positive | + |
| F_2_-84 | 27 | 26.49 | 0.51 | Positive | + |
| F_2_-85 | 25.2 | 24.74 | 0.46 | Positive | + |
| F_2_-86 | Undetermined | 28.08 |  | Negative | - |
| F_2_-87 | 24.75 | 24.74 | 0.01 | Positive | + |
| F_2_-88 | 21.08 | 21.8 | -0.72 | Positive | + |
| F_2_-89 | 21.54 | 21.37 | 0.17 | Positive | + |
| F_2_-90 | 25.68 | 26.01 | -0.33 | Positive | + |
| F_2_-91 | 26.21 | 25.98 | 0.23 | Positive | + |
| F_2_-92 | Undetermined | 25.64 |  | Negative | - |
| F_2_-93 | 27.17 | 26.59 | 0.58 | Positive | + |
| F_2_-94 | 25.37 | 26.03 | -0.66 | Positive | + |
| F_2_-95 | 26.63 | 26.32 | 0.31 | Positive | + |
| F_2_-96 | 27.24 | 26.39 | 0.85 | Positive | + |
| F_2_-97 | 27.44 | 26.54 | 0.9 | Positive | + |
| F_2_-98 | 24.98 | 25.87 | -0.89 | Positive | + |
| F_2_-99 | Undetermined | 28.32 |  | Negative | - |
| F_2_-100 | 28.21 | 28.17 | 0.04 | Positive | + |
| F_2_-101 | Undetermined | 26.32 |  | Negative | - |
| F_2_-102 | 26 | 26.66 | -0.66 | Positive | + |
| F_2_-103 | 26.95 | 26.9 | 0.05 | Positive | + |
| F_2_-104 | 25.37 | 26.11 | -0.74 | Positive | + |
| F_2_-105 | 26.54 | 26.13 | 0.41 | Positive | + |
| F_2_-106 | 25.65 | 25.74 | -0.09 | Positive | + |
| F_2_-107 | 25.28 | 25.2 | 0.08 | Positive | + |
| F_2_-108 | 26.63 | 26.29 | 0.34 | Positive | + |
| F_2_-109 | 29.09 | 29.16 | -0.07 | Positive | + |
| F_2_-110 | 25.8 | 25.71 | 0.09 | Positive | + |
| F_2_-111 | 26.04 | 25.51 | 0.53 | Positive | + |
| F_2_-112 | Undetermined | 27.19 |  | Negative | - |
| F_2_-113 | 28.35 | 27.54 | 0.81 | Positive | + |
| F_2_-114 | 28.81 | 27.89 | 0.92 | Positive | + |
| F_2_-115 | 25.23 | 25.99 | -0.76 | Positive | + |
| F_2_-116 | Undetermined | 27.28 |  | Negative | - |
| F_2_-117 | 27.52 | 27.61 | -0.09 | Positive | + |
| F_2_-118 | 27.02 | 27.64 | -0.62 | Positive | + |
| F_2_-119 | 27.75 | 28.72 | -0.97 | Positive | + |
| F_2_-120 | 25.98 | 26.93 | -0.95 | Positive | + |
| 40-3-2-1 | 22.23 | 22.32 | -0.09 | Positive | + |
| 40-3-2-2 | 25.33 | 25.52 | -0.19 | Positive | + |
| 40-3-2-3 | 26.15 | 26.58 | -0.43 | Positive | + |
| 40-3-2-4 | 22.57 | 23.21 | -0.64 | Positive | + |
| 40-3-2-5 | 24.85 | 24.93 | -0.08 | Positive | + |
| NJW-1 | Undetermined | 22.28 |  | Negative | - |
| NJW-2 | Undetermined | 23.43 |  | Negative | - |
| NJW-3 | Undetermined | 22.66 |  | Negative | - |
| NJW-4 | Undetermined | 24.47 |  | Negative | - |
| NJW-5 | Undetermined | 25.38 |  | Negative | - |

Positive results: exogenous Ct value <35; Ct value of lectin gene <35.

Negative results: no exogenous amplification, no Ct value, Ct value of lectin gene <35.

Reproducibility test for suspicious samples: For samples with exogenous Ct value >35 and Ct value <35, the DNA was re-purified and confirmed by amplification.

“+” means that the epsps protein was detected when the sample was tested with a test strip.

“-” means that the epsps protein was not detected when the sample was tested with a test strip.

**Table S2** Digital PCR determination results and genotype identification results

| **Material number** | **The number of copies** | | | | **DBN/Lectin** **ratio** | **Conclusion** |
| --- | --- | --- | --- | --- | --- | --- |
|  | **GTS40-3-2** | **Average** | **Lectin** | **Average** |  |  |
| F_2_-1 | 262 | 268 | 449 | 452.5 | 0.59 | Heterozygote |
|  | 274 |  | 456 |  |  |  |
| F_2_-2 | 35.4 | 35.2 | 49.6 | 51.8 | 0.68 | Heterozygote |
|  | 35 |  | 54 |  |  |  |
| F_2_-3 | 81.8 | 81.4 | 61.1 | 64.15 | 1.27 | Positive homozygote |
|  | 81 |  | 67.2 |  |  |  |
| F_2_-5 | 267 | 265.5 | 438 | 440.5 | 0.60 | Heterozygote |
|  | 264 |  | 443 |  |  |  |
| F_2_-7 | 36.2 | 36.75 | 34.4 | 36.3 | 1.01 | Positive homozygote |
|  | 37.3 |  | 38.2 |  |  |  |
| F_2_-8 | 13 | 12.55 | 15.9 | 15.35 | 0.82 | Positive homozygote |
|  | 12.1 |  | 14.8 |  |  |  |
| F_2_-9 | 7.6 | 8.7 | 12.7 | 14.45 | 0.60 | Heterozygote |
|  | 9.8 |  | 16.2 |  |  |  |
| F_2_-10 | 6.1 | 6.25 | 4.7 | 5 | 1.25 | Positive homozygote |
|  | 6.4 |  | 5.3 |  |  |  |
| F_2_-11 | 15.6 | 16.1 | 12.8 | 12.25 | 1.31 | Positive homozygote |
|  | 16.6 |  | 11.7 |  |  |  |
| F_2_-12 | 269 | 271.5 | 221 | 216 | 1.26 | Positive homozygote |
|  | 274 |  | 211 |  |  |  |
| F_2_-14 | 116 | 113.5 | 192 | 194 | 0.59 | Heterozygote |
|  | 111 |  | 196 |  |  |  |
| F_2_-15 | 192 | 193 | 307 | 309.5 | 0.62 | Heterozygote |
|  | 194 |  | 312 |  |  |  |
| F_2_-16 | 214 | 220 | 350 | 356.5 | 0.62 | Heterozygote |
|  | 226 |  | 363 |  |  |  |
| F_2_-18 | 146 | 148.5 | 234 | 235.5 | 0.63 | Heterozygote |
|  | 151 |  | 237 |  |  |  |
| F_2_-20 | 229 | 226.5 | 391 | 389.5 | 0.58 | Heterozygote |
|  | 224 |  | 388 |  |  |  |
| F_2_-21 | 271 | 275 | 531 | 531 | 0.52 | Heterozygote |
|  | 279 |  | 531 |  |  |  |
| F_2_-23 | 147 | 148 | 248 | 247 | 0.60 | Heterozygote |
|  | 149 |  | 246 |  |  |  |
| F_2_-24 | 21.3 | 21 | 30.5 | 30.15 | 0.70 | Heterozygote |
|  | 20.7 |  | 29.8 |  |  |  |
| F_2_-25 | 164 | 169 | 278 | 270 | 0.63 | Heterozygote |
|  | 174 |  | 262 |  |  |  |
| F_2_-26 | 297 | 294.5 | 481 | 487 | 0.60 | Heterozygote |
|  | 292 |  | 493 |  |  |  |
| F_2_-27 | 205 | 203 | 364 | 366 | 0.55 | Heterozygote |
|  | 201 |  | 368 |  |  |  |
| F_2_-28 | 92 | 91.5 | 79.5 | 77.25 | 1.18 | Positive homozygote |
|  | 91 |  | 75 |  |  |  |
| F_2_-31 | 39.2 | 40.6 | 64.5 | 63.75 | 0.64 | Heterozygote |
|  | 42 |  | 63 |  |  |  |
| F_2_-32 | 748 | 752.5 | 674 | 665.5 | 1.13 | Positive homozygote |
|  | 757 |  | 657 |  |  |  |
| F_2_-33 | 624 | 614 | 587 | 586 | 1.05 | Positive homozygote |
|  | 604 |  | 585 |  |  |  |
| F_2_-34 | 373 | 379 | 646 | 653 | 0.58 | Heterozygote |
|  | 385 |  | 660 |  |  |  |
| F_2_-36 | 227 | 233 | 420 | 416.5 | 0.56 | Heterozygote |
|  | 239 |  | 413 |  |  |  |
| F_2_-37 | 462 | 457.5 | 763 | 799.5 | 0.57 | Heterozygote |
|  | 453 |  | 836 |  |  |  |
| F_2_-39 | 545 | 547 | 527 | 519.5 | 1.05 | Positive homozygote |
|  | 549 |  | 512 |  |  |  |
| F_2_-40 | 746 | 745 | 677 | 681 | 1.09 | Positive homozygote |
|  | 744 |  | 685 |  |  |  |
| F_2_-41 | 334 | 338 | 311 | 310.5 | 1.09 | Positive homozygote |
|  | 342 |  | 310 |  |  |  |
| F_2_-42 | 36.4 | 35.15 | 24.9 | 24.8 | 1.42 | Positive homozygote |
|  | 33.9 |  | 24.7 |  |  |  |
| F_2_-43 | 83 | 81 | 122 | 124 | 0.65 | Heterozygote |
|  | 79 |  | 126 |  |  |  |
| F_2_-44 | 229 | 231 | 450 | 451.5 | 0.51 | Heterozygote |
|  | 233 |  | 453 |  |  |  |
| F_2_-45 | 95 | 92.5 | 156 | 160 | 0.58 | Heterozygote |
|  | 90 |  | 164 |  |  |  |
| F_2_-47 | 357 | 354 | 377 | 379.5 | 0.93 | Positive homozygote |
|  | 351 |  | 382 |  |  |  |
| F_2_-48 | 235 | 231.5 | 290 | 287 | 0.81 | Positive homozygote |
|  | 228 |  | 284 |  |  |  |
| F_2_-50 | 248 | 253 | 386 | 387.5 | 0.65 | Heterozygote |
|  | 258 |  | 389 |  |  |  |
| F_2_-52 | 427 | 424.5 | 331 | 334 | 1.27 | Positive homozygote |
|  | 422 |  | 337 |  |  |  |
| F_2_-53 | 42 | 40.5 | 71 | 72.75 | 0.56 | Heterozygote |
|  | 39 |  | 74.5 |  |  |  |
| F_2_-54 | 145 | 142.5 | 278 | 276 | 0.52 | Heterozygote |
|  | 140 |  | 274 |  |  |  |
| F_2_-55 | 109 | 111.5 | 108 | 106 | 1.05 | Positive homozygote |
|  | 114 |  | 104 |  |  |  |
| F_2_-56 | 460 | 456 | 377 | 383 | 1.19 | Positive homozygote |
|  | 452 |  | 389 |  |  |  |
| F_2_-58 | 61 | 65 | 117 | 121 | 0.54 | Heterozygote |
|  | 69 |  | 125 |  |  |  |
| F_2_-59 | 220 | 218 | 360 | 365 | 0.60 | Heterozygote |
|  | 216 |  | 370 |  |  |  |
| F_2_-60 | 284 | 281.5 | 259 | 263 | 1.07 | Positive homozygote |
|  | 279 |  | 267 |  |  |  |
| F_2_-62 | 450 | 445.5 | 416 | 413.5 | 1.08 | Positive homozygote |
|  | 441 |  | 411 |  |  |  |
| F_2_-63 | 109 | 106 | 206 | 211 | 0.50 | Heterozygote |
|  | 103 |  | 216 |  |  |  |
| F_2_-64 | 67 | 64.5 | 94 | 96.5 | 0.67 | Heterozygote |
|  | 62 |  | 99 |  |  |  |
| F_2_-65 | 7.9 | 8.7 | 6.8 | 6.15 | 1.41 | Positive homozygote |
|  | 9.5 |  | 5.5 |  |  |  |
| F_2_-66 | 331 | 334.5 | 238 | 247.5 | 1.35 | Positive homozygote |
|  | 338 |  | 257 |  |  |  |
| F_2_-68 | 325 | 332 | 264 | 271.5 | 1.22 | Positive homozygote |
|  | 339 |  | 279 |  |  |  |
| F_2_-69 | 288 | 283 | 460 | 463.5 | 0.61 | Heterozygote |
|  | 278 |  | 467 |  |  |  |
| F_2_-70 | 161 | 159 | 225 | 229 | 0.69 | Heterozygote |
|  | 157 |  | 233 |  |  |  |
| F_2_-71 | 20 | 20.8 | 22 | 21 | 0.99 | Positive homozygote |
|  | 21.6 |  | 20 |  |  |  |
| F_2_-72 | 288 | 279 | 421 | 426 | 0.65 | Heterozygote |
|  | 270 |  | 431 |  |  |  |
| F_2_-73 | 31 | 33.5 | 65 | 62 | 0.54 | Heterozygote |
|  | 36 |  | 59 |  |  |  |
| F_2_-76 | 334 | 331.5 | 366 | 369.5 | 0.90 | Positive homozygote |
|  | 329 |  | 373 |  |  |  |
| F_2_-77 | 159 | 157 | 173 | 174.5 | 0.90 | Positive homozygote |
|  | 155 |  | 176 |  |  |  |
| F_2_-78 | 28 | 30 | 59 | 57.7 | 0.52 | Heterozygote |
|  | 32 |  | 56.4 |  |  |  |
| F_2_-80 | 147 | 150 | 301 | 295 | 0.51 | Heterozygote |
|  | 153 |  | 289 |  |  |  |
| F_2_-81 | 73 | 76 | 148 | 146.5 | 0.52 | Heterozygote |
|  | 79 |  | 145 |  |  |  |
| F_2_-82 | 288 | 285.5 | 279 | 278 | 1.03 | Positive homozygote |
|  | 283 |  | 277 |  |  |  |
| F_2_-83 | 105 | 107.5 | 203 | 200.5 | 0.54 | Heterozygote |
|  | 110 |  | 198 |  |  |  |
| F_2_-84 | 93 | 92 | 162 | 164 | 0.56 | Heterozygote |
|  | 91 |  | 166 |  |  |  |
| F_2_-85 | 308 | 314.5 | 499 | 506 | 0.62 | Heterozygote |
|  | 321 |  | 513 |  |  |  |
| F_2_-87 | 23 | 24 | 22 | 22.75 | 1.05 | Positive homozygote |
|  | 25 |  | 23.5 |  |  |  |
| F_2_-88 | 211 | 214 | 197 | 193.5 | 1.11 | Positive homozygote |
|  | 217 |  | 190 |  |  |  |
| F_2_-89 | 243 | 241 | 327 | 328 | 0.73 | Heterozygote |
|  | 239 |  | 329 |  |  |  |
| F_2_-90 | 237 | 236 | 344 | 343.5 | 0.69 | Heterozygote |
|  | 235 |  | 343 |  |  |  |
| F_2_-91 | 491 | 493 | 421 | 423 | 1.17 | Positive homozygote |
|  | 495 |  | 425 |  |  |  |
| F_2_-93 | 368 | 364 | 297 | 300 | 1.21 | Positive homozygote |
|  | 360 |  | 303 |  |  |  |
| F_2_-94 | 47 | 45.5 | 81 | 79.5 | 0.57 | Heterozygote |
|  | 44 |  | 78 |  |  |  |
| F_2_-95 | 155 | 154.5 | 158 | 157.5 | 0.98 | Positive homozygote |
|  | 154 |  | 157 |  |  |  |
| F_2_-96 | 143 | 145 | 261 | 260 | 0.56 | Heterozygote |
|  | 147 |  | 259 |  |  |  |
| F_2_-97 | 414 | 402 | 369 | 361.5 | 1.11 | Positive homozygote |
|  | 390 |  | 354 |  |  |  |
| F_2_-98 | 14.2 | 14.6 | 16 | 16.5 | 0.88 | Positive homozygote |
|  | 15 |  | 17 |  |  |  |
| F_2_-100 | 39 | 38 | 63 | 62.5 | 0.61 | Heterozygote |
|  | 37 |  | 62 |  |  |  |
| F_2_-102 | 249 | 242.5 | 466 | 460 | 0.53 | Heterozygote |
|  | 236 |  | 454 |  |  |  |
| F_2_-103 | 330 | 323 | 441 | 440 | 0.73 | Heterozygote |
|  | 316 |  | 439 |  |  |  |
| F_2_-104 | 453 | 451.5 | 481 | 479 | 0.94 | Positive homozygote |
|  | 450 |  | 477 |  |  |  |
| F_2_-105 | 406 | 409.5 | 289 | 292.5 | 1.40 | Positive homozygote |
|  | 413 |  | 296 |  |  |  |
| F_2_-106 | 115 | 112 | 190 | 193 | 0.58 | Heterozygote |
|  | 109 |  | 196 |  |  |  |
| F_2_-107 | 36 | 35.5 | 57 | 57 | 0.62 | Heterozygote |
|  | 35 |  | 57 |  |  |  |
| F_2_-108 | 148 | 145.5 | 213 | 214 | 0.68 | Heterozygote |
|  | 143 |  | 215 |  |  |  |
| F_2_-109 | 177 | 179 | 299 | 303.5 | 0.59 | Heterozygote |
|  | 181 |  | 308 |  |  |  |
| F_2_-110 | 353 | 351 | 271 | 270.5 | 1.30 | Positive homozygote |
|  | 349 |  | 270 |  |  |  |
| F_2_-111 | 65 | 65 | 105 | 103 | 0.63 | Heterozygote |
|  | 65 |  | 101 |  |  |  |
| F_2_-113 | 197 | 195 | 338 | 343.5 | 0.57 | Heterozygote |
|  | 193 |  | 349 |  |  |  |
| F_2_-114 | 94 | 95 | 171 | 173 | 0.55 | Heterozygote |
|  | 96 |  | 175 |  |  |  |
| F_2_-115 | 294 | 295.5 | 280 | 275.5 | 1.07 | Positive homozygote |
|  | 297 |  | 271 |  |  |  |
| F_2_-117 | 55 | 53 | 84 | 82 | 0.65 | Heterozygote |
|  | 51 |  | 80 |  |  |  |
| F_2_-118 | 51 | 48.5 | 90 | 92 | 0.53 | Heterozygote |
|  | 46 |  | 94 |  |  |  |
| F_2_-119 | 73 | 75.5 | 109 | 107.5 | 0.70 | Heterozygote |
|  | 78 |  | 106 |  |  |  |
| F_2_-120 | 369 | 363.5 | 378 | 381.5 | 0.95 | Positive homozygote |
|  | 358 |  | 385 |  |  |  |
| 40-3-2-1 | 324 | 326.5 | 310 | 313.5 | 1.04 | Positive homozygote |
|  | 329 |  | 317 |  |  |  |
| 40-3-2-2 | 342 | 344.5 | 295 | 291.5 | 1.18 | Positive homozygote |
|  | 347 |  | 288 |  |  |  |
| 40-3-2-3 | 198 | 193.5 | 179 | 176 | 1.10 | Positive homozygote |
|  | 189 |  | 173 |  |  |  |
| 40-3-2-4 | 268 | 271 | 255 | 251 | 1.08 | Positive homozygote |
|  | 274 |  | 247 |  |  |  |
| 40-3-2-5 | 369 | 372.5 | 362 | 360 | 1.03 | Positive homozygote |
|  | 376 |  | 358 |  |  |  |
| NJW-1 | 0 | 0 | 342 | 339 | 0.00 | Negative |
|  | 0 |  | 336 |  |  |  |
| NJW-2 | 0 | 0 | 179 | 182.5 | 0.00 | Negative |
|  | 0 |  | 186 |  |  |  |
| NJW-3 | 0 | 0 | 233 | 237 | 0.00 | Negative |
|  | 0 |  | 241 |  |  |  |
| NJW-4 | 0 | 0 | 369 | 363.5 | 0.00 | Negative |
|  | 0 |  | 358 |  |  |  |
| NJW-5 | 0 | 0 | 274 | 279.5 | 0.00 | Negative |
|  | 0 |  | 285 |  |  |  |

The theoretical value of the copy number ratio of homozygotes was 1 and that of heterozygotes was 0.5.
